# Early Neurological Assessment in Infants with Hypoxic Ischemic Encephalopathy Treated with Therapeutic Hypothermia

**DOI:** 10.3390/jcm8081247

**Published:** 2019-08-18

**Authors:** Domenico M. Romeo, Sarah Bompard, Francesca Serrao, Giuseppina Leo, Gianpaolo Cicala, Chiara Velli, Francesca Gallini, Francesca Priolo, Giovanni Vento, Eugenio Mercuri

**Affiliations:** 1 Pediatric Neurology Unit, Fondazione Policlinico Universitario A. Gemelli IRCCS, 00168 Rome, Italy; 2 Pediatric Neurology Unit, Università Cattolica del Sacro Cuore Roma, 00168 Rome, Italy; 3 Neonatal Intensive Care Unit, Fondazione Policlinico Universitario A. Gemelli IRCCS, 00168 Rome, Italy

**Keywords:** hypoxic ischemic encephalopathy, hypothermia, Hammersmith Infant Neurological Examination, outcome

## Abstract

Early neurological assessment in infants with hypoxic ischemic encephalopathy (HIE) treated with hypothermia has not been systematically explored. The aims of the present study were to assess whether the Hammersmith Infant Neurological Examination (HINE) is a good tool to predict later neurodevelopmental outcomes at 2 year from birth in this population of infants. A total of 41 term born infants with HIE treated with hypothermia performed the HINE at 12 months and a neurodevelopmental assessment at 24 months. All the infants who had a global HINE score between 67 and 78 were able to walk independently at 2 years and reported a normal developmental quotient; language disorders were observed in a limited number of infants. HINE scores <67 were always associated with motor impairment. In conclusion, the HINE confirms its role as one of the early neurological examination tools for the diagnosis of high risk infants, even in infants with HIE treated with hypothermia. These results can be useful for clinicians involved in the follow up of these infants for early identification of motor disabilities and in planning appropriate intervention.

## 1. Introduction

Neonatal hypoxic ischemic encephalopathy (HIE) remains one of the leading causes of newborn mortality and long term disability worldwide, as it occurs in 3–5 out of 1000 live births [1,2,3]. In the last 20 years, therapeutic hypothermia (TH) has been used to treat newborns with HIE and it is now considered to be the standard of care for this population of neonates [1,2,3]; although several randomized controlled trials have demonstrated the efficacy of TH in the reduction in the risk of death or disability at pre-school and school age, a quote of 30–50% of these neonates still suffer major disabilities [1,4,5,6]. The possibility to determine which patients are at highest risk of neurological impairment is limited [6,7] and most of the neurophysiological tests used to assess prognosis had a good power prediction in term of normal/abnormal outcome only, but did not add information on the prognosis in term of type and severity of the functional motor outcome; furthermore, some of these tests, such as comprehensive neurophysiological assessment and neuroimaging, may be limited to specialist centers [7].

Neurological assessment is one of the clinical tools used to monitor development in infants that are at risk of developing neurodevelopmental disabilities [8,9]. In recent years, the Hammersmith Infant Neurological Examination (HINE) has been identified as one of of the best and simplest neurological examinations for the early diagnosis of neurological impairment in both low and high-risk infants, as it can even be easily performed by inexperienced staff [8,9,10,11]. The assessment includes several aspects of neurological functions, including cranial nerve assessment, posture, movements, tone, reflexes, and behavior, and it also provides additional information on the type and severity of the overall disability, which is not limited to motor impairment [9].

In infants with HIE, this neurologic examination provided additional prognostic information on the severity of the functional motor outcome at 2 and 4 years, showing a good correlation with the MRI performed during the neonatal period [12]. However, the role of the HINE infants with HIE in the hypothermia era has not been systematically investigated. This data could be of interest to identify those infants at risk of neuromotor impairment and to start an early rehabilitation program.

The aims of the present study was therefore to test the hypothesis that the HINE could be used to predict later neurodevelopmental outcomes at 2 year from birth in newborns with HIE treated with TH.

## 2. Materials and Methods

The infants described in this study were part of a cohort of term infants who were born with perinatal hypoxic-ischemic brain injury and admitted to the Neonatal Unit of the Fondazione Policlinico Gemelli between January 2015 and June 2017 and consecutively enrolled in a follow-up prospective research program. 

For the purpose of this study, infants with ≥36 weeks gestation, with signs of encephalopathy after 10 min after birth but before 6 h, were recruited if they had one of the following signs suggesting intrapartum hypoxia: (i) a pH ≤ 7.0 or a base deficit ≥16 mmoL/L in the first hour of life on cord or infant arterial blood, or (ii) An abnormal intra-partum course (e.g., abnormal fetal heart rate, cord prolapse, uterine rupture, maternal haemorrhage/trauma/seizures/cardiorespiratory arrest; shoulder dystocia; meconium-stained liquor or prolonged second stage) and either a 10 min Apgar score ≤5 or continued respiratory support at 10 min. 

Once these criteria were met, all infants underwent a standardized neurologic examination [1,13]. Infants were candidates for the study when encephalopathy or seizures were present. The encephalopathy was graded as mild (hyperalert, normal tone and activity, exaggerated moro, normal autonomic function), moderate (lethargic, decreased activity, distil flexion, hypotonia, weak primitive reflexes, constricted pupils, bradycardia or periodic breathing), or severe (stupor/coma, decerebrate posture, absent spontaneous activity, flaccid, absent primitive reflexes, non-reactive pupils or apnoea). The grade with the most corresponding signs was assigned but if signs were equally distributed, the grade was based on the level of consciousness. Infants with seizures were graded as “moderate” unless severe signs predominated. These assessments were performed within 1 h. 

Exclusion criteria were a completely normal neurological exam; inability to enrol at ≤6 h of age; major congenital abnormality, cerebral malformations; neonatal abstinence syndrome; metabolic encephalopathies; a severe growth restriction (birth weight of ≤1800 g); refusal of consent by a parent; moribund infants for whom no further aggressive treatment was planned also were excluded.

Parental permission was obtained in all cases. 

All the infant who met the inclusion criteria and a moderate or severe encephalopathy underwent TH according to the international guideline for 72 h [1]. During TH, infants received routine clinical care, including the monitoring of vital signs and surveillance for organ dysfunction.

A magnetic resonance imaging (MRI) was performed in all the newborns between days 7 to 14 after birth.

### 2.1. Neurodevelopmental Assessment

The Hammersmith Infant Neurological Examination (HINE) was performed at 12 months [9]. An optimality score was obtained by calculating the distribution of the frequency of the scores in the normal population, defining as optimal all the scores found in at least 90% of the cohort. The overall score ranges from a minimum of 0 to a maximum of 78. At 9 and 12 months, a score ≥73 is regarded as optimal, while <73 is sub-optimal [9]. 

A further neurodevelopmental assessment was performed at 2 years; it included a structured neurological examination according to Touwen [14] and a developmental assessment, using the Griffiths Scale of Mental Development [15]. The scale includes five subscales (locomotor, personal–social, hearing and speech, hand and eye co-ordination, and performance) and, for the children older than two, an additional subscale assessing practical reasoning. One of the advantages of this test is that it provides not only a global developmental quotient (DQ) but also subquotients for the individual subscales, allowing us to obtain a profile of abilities in the individual subscales. Results were classified as normal when the DQ were 85 or above, borderline if falling between 84 and 70 and abnormal below 70. 

Cerebral palsy, if present, was classified according to the criteria proposed by Himmelmann et al. [16].

The Ethical Committee of our Institution approved the study. 

### 2.2. Statistical Analysis

The anthropometric variables (weight and gestational age) and HINE scores were reported as median and range. In order to explore the possible influence of gender on birth-weight, gestational age, HINE scores and DQ on Griffitth’ scales, differences in sub-scores and global scores between male and female were analysed by the Mann-Whitney test. Statistical analysis was performed using the “Stata Statistical Software: Release 10” (StataCorp LP, College Station, TX, USA). The level of significance was set at *p* < 0.01. 

## 3. Results

A total of 48 infants were eligible for the study. Seven of them (one deceased during the neonatal period) did not attend one or more of the assessments and were therefore excluded. Forty-one infants represented the final population (12 females, 29 males) with a median birth-weight of 3190 g (range 2040–5000 gr) and a median gestational age of 39 weeks (range 36–41 weeks). There was no difference between males and females on birth-weight or gestational age (*p* > 0.05). 

All the infants performed a MRI scan during the neonatal period (range 7–14 days; median 10 days after birth) with a similar distribution of normal/abnormal scans between males and females. A total of 23 of the 41 infants had normal scans (7 females, 16 males). Of the 18/41 infants with abnormal scans, 8 had minimal basal ganglia and thalami lesions (1 females, 7 males), 4 had moderate white matter lesions (1 female, 3 males), 3 had moderate white matter and basal ganglia and thalami (1 female, 2 males), while 3 had severe basal ganglia and thalami with diffuse white matter (2 females, 1 male). 

### 3.1. Neurological Examination

At 12 months of age, 30 infants reached an optimality score (≥73) and 11 reported a suboptimal score, 7 with scores between 67 and 72, 3 with scores between 40–66 and only one with scores <40. Table 1 reported total and subscale scores in the population. No difference was found between males and females on HINE total and subscale scores (*p* > 0.05).

### 3.2. Neurodevelopmental Assessment at Two Years

Thirty-six infants were neurologically normal, 1 had a diplegia, 2 had quadriplegia, and 2 had quadriplegia with dystonia. Thirty-seven of the 41 infants were able to walk independently. The remaining 4 infants showed severely limited self-mobility and unsupported sitting.

The global DQ of the Griffitths scale was >85 in 37 infants and was abnormal (<70) in the other 4. No difference was found between males and females on global DQ (*p* > 0.05). In the 37 infants with normal DQ, 7 reported a borderline DQ in the hearing and speech subscale (1 female, 6 males), 4 an abnormal DQ in the hearing and speech subscale (4 males), 1 a borderline DQ in the locomotor subscale (1 female) and 1 a border DQ in the personal–social subscale (1 male).

### 3.3. HINE Scores and Outcome

**Figure 1:**
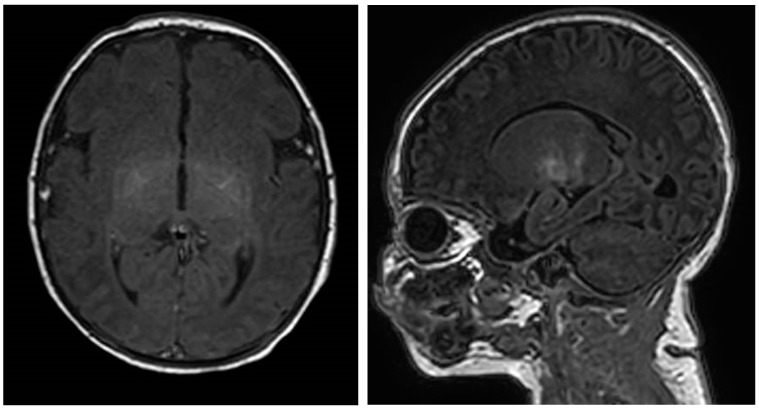
MRI findings in an infant with HINE score >73 and a language delay, Axial and sagittal T1-weighted images show slight increased signal intensity in the lentiform nucleus.

The neurologic scores were optimal in all the children but 3 with normal MRI or moderate white matter lesions and in 6 out of 8 (75%) of the infants with minimal basal ganglia lesions (Table 2, Figure 1). The scores were suboptimal in all the others and were very low in the infants with severe basal ganglia lesions associated with white matter lesions and intermediate in the ones with minimal basal ganglia lesions, moderate basal ganglia and white matter lesions.

Table 3 shows details of the optimality scores and neurodevelopmental assessment at 2 years. 

Thirty infants had optimal HINE scores: all walked independently by the age of 2 years and had a normal global DQ. Three of them had abnormal DQ in the hearing and speech subscale (1 with normal and 2 with Minimal basal ganglia lesions at MRI scans) (Figure 1).

Seven infants had suboptimal HINE scores (above 67): all were able to walk independently by the age of 2 years and had a normal DQ. One of the 7 had abnormal DQ in the hearing and speech subscale (normal MRI scan) and another one a borderline DQ in the locomotor subscale and a mild form of diplegia (moderate white matter and basal ganglia lesion at MRI scans). 

Three infants had HINE scores between 66 and 40: all of these infants had motor impairment and could not sit unsupported at 2. 

The only one infant with a HINE score <40 showed severe motor impairment with incomplete head control.

## 4. Discussion

This is the first study reporting early neurological assessment in infants with HIE treated with hypothermia. For the purpose of this study, we used the HINE as it has been reported to have a good predictive power for motor outcome in both preterm and term born infants [8,9,10,11,12]. 

In pre-hypothermia era, approximately 40% of infants with HIE had suboptimal scores on the HINE that was performed at 9–14 months [12], with an overall good association between HINE scores, patterns of MRI lesion, and severity of functional motor impairment at 2 and 4 years. The results of the present study confirm the role of the HINE in assessing high-risk infants, even in those who underwent TH after birth. 

The results obtained in our cohort of infants treated with hypothermia showed better HINE scores than those previously reported in a similar population of infants with HIE without TH [12]. The HINE data mirrored an overall less severe pattern of brain MRI findings and better neurodevelopmental outcome. This data is in agreement with previous studies showing a reduction in cerebral lesions on brain MRI [17], better neurodevelopmental and motor outcome in cooled HIE infants compared to not treated HIE ones [1,2,3,4,5,6,7].

In the present study, only 27% of the infants reported suboptimal HINE scores. The magnitude of the scores were often related to the brain MRI findings. The HINE scores were always optimal or slightly sub-optimal (>67) in the infants with normal neonatal MRI or moderate white matter lesions. At the other extreme, the lowest HINE scores were always associated with very severe lesions such as severe basal ganglia and diffuse white matter lesions. Infants with minimal basal ganglia or with moderate white matter and basal ganglia lesions had more variable scores with both optimal and sub-optimal scores. In a single case, MRI reported a minimal basal ganglia lesions only with a sub-optimal score of <67; this infant showed spastic quadriplegia at 2 years.

The HINE scores were also related to the neurodevelopmental outcome at 2 years. Only one infant had a score <40 and showed a severe form of spastic-dystonic quadriplegia without head control. Infants with sub-optimal scores <67 were always associated with motor impairment and unsupported sitting at 2 years. Infants with suboptimal scores but above 67 were always associated with the ability to walk independently at 2 years, only in one case associated with a mild form of diplegia. All the infants with optimal scores had a normal motor outcome. These findings are in agreement with previous published studies correlating the patterns of lesions on MRI with the HINE optimality scores and motor outcome in high-risk term born infants [9,12,18]. The main difference between the studies is that in the previous studies reporting HIE in non-cooled infants, the pattern of brain lesions was much more severe. It is of interest that in our cohort we found a number of infants with mild basal ganglia lesions, with the typical involvement of the lentiform nuclei but with less severe changes and an overall less diffuse signal abnormality. Further analysis of these findings are ongoing to better classify the severity of the MRI changes and ascertain whether treatment with hypothermia may not only result in a reduction of lesions but possibly also in milder changes in signal intensity that may be associated with a more favourable outcome. A longer follow up of our patients will also help to better correlate our findings with previous studies who had a longer outcome (4 years) compared to ours (24 months). Although the risk of developing a severe cerebral palsy after the age of 2 years is unlikely, one cannot exclude the possibility that some of these infants may show minor neurological signs that may not be detected at 24 months.

The possibility to relate the HINE scores to the Griffith’s scales allowed further considerations. It is of interest that among the 37 infants with normal neurodevelopmental outcome, 11 had borderline (*n* = 7) or abnormal (*n* = 4) DQ on the hearing and speech subscale. All of them had a normal MRI or minimal basal ganglia lesions, optimal HINE scores in 73% and suboptimal but above 67 in the other 27%. These results are consistent with the few studies investigating language development in children with HIE post-TH, also showing significantly lower scores in expressive language even in the absence of more serious cognitive or motor difficulties or with minimal or no MRI lesions [19,20]. The interpretation of this finding is difficult for several reasons. First, the Griffiths scales at two years provide an indication of both hearing and speech but are not specifically designed for assessing different aspects of language. Other tools, such as the MacArthur-Bates Communicative Development Inventories [21], providing early language skills of infants and toddlers, including comprehension, vocabulary, grammatical skills, and nonverbal gestures/actions may have helped to better identify infants at risk of language impairment. A longer follow up using more appropriate and specific scales assessing language in older infants would further help to establish the possible persistence of the difficulties observed at 2 years. Another possible explanation may come from recent morphometric MRI study analysis suggesting that an underdevelopment of cortical gray matter and subcortical white matter at 6 months of age, presumed to be due to an early hypoxic-ischemic insult, may be associated with poorer language skills in early childhood [19].

In the present study there was an overall higher number of males then females; this is not surprising, as the male sex is recognized to be a risk factor for neonatal HIE during the perinatal period even in those treated with hypothermia [22]; males are reported to be twice more likely to experience prenatal anoxia, hemorrhage, and infection and ischemic injury appears to be more common in boys regardless of lesion types [22]; sex differences in inflammatory responses, microglial activation, metabolic profile, brain structure and plasticity have been suggested to have a role [22,23]. It is of interest that in our cohort, males appeared to be at higher risk of lower scores on the hearing and speech subscale, in agreement with other studies also reporting that girls had better performance than boys on the expressive language [19,20].

In conclusions our results suggest that the HINE could be used to assess infants with HIE, even in the hypothermia era. These results are very promising but should be interpreted with caution because of the relatively small number of patients included. Larger cohorts and a longer follow up are needed to confirm our preliminary results and to allow a more detailed correlation between clinical and neuroradiological assessments. These findings: even if preliminary, can be useful for clinicians involved in the follow up of infants with HIE treated with hypothermia. Suboptimal HINE scores may help, especially if associated with severe brain lesions, to early identify patients who may benefit from early appropriate intervention. On the other hand, the presence of optimal HINE scores and normal brain MRI or minimal changes will help to reassure parents about a lower risk of developing severe squealae.

## Figures and Tables

**Table 1:** HINE scores in total population.

	Cranial Nerve	Spontaneous Mot.	Posture	Tone	Reflexes	Total
Median	15	6	17	22	14	73
Range	3–15	1–6	4–18	3–24	2–15	13–78

**Table 2:** HINE scores and neonatal MRI findings.

MRI Finding	Median Score	Range of the Scores
Normal (*n* = 23)	76	68–78
Moderate WM (*n* = 4)	77	75–78
Minimal BG (*n* = 8)	76	45–77
Moderate WM and BG (*n* = 3)	71	68.5–72
Severe BG and diffuse WM (*n* = 3)	43.5	13–49

**Table 3:** Optimality scores and MRI findings: Correlation with outcome at 2 years.

MRI Finding	Optimal (HINE Score ≥73)	Suboptimal (HINE Score 67–73)	Suboptimal (HINE Score 40–66)	Suboptimal (HINE Score <40)
Normal (*n* = 23)	❍❍❍❍❍❍❍❍❍❍❍❍❍❍❍❍❍❍❍◊	❍❍◊		
Moderate WM (*n* = 4)	❍❍❍❍			
Minimal BG (*n* = 8)	❍❍❍❍◊◊	❍	●	
Moderate WM and BG (*n* = 3)		❍❍●		
Severe BG and diffuse WM (*n* = 3)			●●	●

BG = basal ganglia; WM = white matter; ❍ = normal outcome; ◊ = language delay; ● = cerebral palsy.
